# Association and *cis*-mQTL analysis of variants in *CHRNA3-A5, CHRNA7, CHRNB2*, and *CHRNB4* in relation to nicotine dependence in a Chinese Han population

**DOI:** 10.1038/s41398-018-0130-x

**Published:** 2018-04-18

**Authors:** Qiang Liu, Haijun Han, Maiqiu Wang, Yinghao Yao, Li Wen, Keran Jiang, Yunlong Ma, Rongli Fan, Jiali Chen, Kunkai Su, Zhongli Yang, Wenyan Cui, Wenji Yuan, Xianzhong Jiang, Jingjing Li, Thomas J. Payne, Jundong Wang, Ming D. Li

**Affiliations:** 1 State Key Laboratory for Diagnosis and Treatment of Infectious Diseases, The First Affiliated Hospital, Collaborative Innovation Center for Diagnosis and Treatment of Infectious Diseases, Zhejiang University School of Medicine, Hangzhou, China; 2 ACT Center for Tobacco Treatment, Education and Research, Department of Otolaryngology and Communicative Sciences, University of Mississippi Medical Center, Jackson, MS USA; 3 Shanxi Key Laboratory of Environmental Veterinary Medicine, Shanxi Agricultural University, Taigu, China; 4 Research Center for Air Pollution and Health, Zhejiang University, Hangzhou, China; 5 Institute of Neuroimmune Pharmacology, Seton Hall University, South Orange, NJ USA

## Abstract

Nicotine dependence (ND) is a worldwide health problem. Numerous genetic studies have demonstrated a significant association of variants in nicotinic acetylcholine receptors (nAChRs) with smoking behaviors. However, most of these studies enrolled only subjects of European or African ancestry. In addition, although an increasing body of evidence implies a causal connection of single-nucleotide polymorphisms (SNPs) and epigenetic regulation of gene expression, few studies of this issue have been reported. In this study, we performed both association and interaction analysis for 67 SNPs in *CHRNA3-A5, CHRNA7, CHRNB2*, and *CHRNB4* with ND in a Chinese Han population (*N* = 5055). We further analyzed *cis*-mQTL for the three most significant SNPs and 5580 potential methylation loci within these target gene regions. Our results indicated that the SNPs rs1948 and rs7178270 in *CHRNB4* and rs3743075 in *CHRNA3* were significantly associated with the Fagerström Test for Nicotine Dependence (FTND) score (*p* = 6.6 × 10^−5^; *p* = 2.0 × 10^−4^, and *p* = 7.0 × 10^−4^, respectively). Haplotype-based association analysis revealed that two major haplotypes, T-G and C-A, formed by rs3743075–rs3743074 in *CHRNA3*, and other two major haplotypes, A-G-C and G-C-C, formed by rs1948–rs7178270–rs17487223 in *CHRNB4*, were significantly associated with the FTND score (*p* ≤ 8.0 × 10^−4^). Further, we found evidence for the presence of significant interaction among variants within *CHRNA3/B4/A5, CHRNA4/B2/A5*, and *CHRNA7* in affecting ND, with corresponding *p* values of 5.8 × 10^−6^, 8.0 × 10^−5^, and 0.012, respectively. Finally, we identified two CpG sites (CpG_2975 and CpG_3007) in *CHRNA3* that are significantly associated with three *cis*-mQTL SNPs (rs1948, rs7178270, rs3743075) in the *CHRNA5/A3/B4* cluster (*p* ≤ 1.9 × 10^−6^), which formed four significant CpG–SNP pairs in our sample. Together, we revealed at least three novel SNPs in *CHRNA3* and *CHRNB4* to be significantly associated with the FTND score. Further, we showed that these significant variants contribute to ND via two methylated sites, and we demonstrated significant interaction affecting ND among variants in *CHRNA5/A3/B4*, *CHRNA7*, and *CHRNA4/B2/A5*. In sum, these findings provide robust evidence that SNPs in nAChR genes convey a risk of ND in the Chinese Han population.

## Introduction

Tobacco smoking is a major public health problem that causes nearly 6 million deaths worldwide every year.^1^ Because of the lack of effective treatment for smoking addiction, the addictive properties of nicotine in tobacco smoke, and lack of awareness of the consequences of smoking in many regions, the worldwide death toll caused by cigarette smoking might well reach 10 million annually by 2020.^2^ Although many developed countries have implemented regulations and laws against tobacco smoking, which have led to dramatic reductions in smoking during recent years, smoking remains a significant issue in many developing countries, especially in Asia.^3,4^ For example, the prevalence of smoking in Chinese men aged 15 or older is an estimated 52.1%,^5^ meaning that male Chinese smokers account for almost one of third of the total number of smokers in the world.^6^

Although environmental factors play an important role in nicotine dependence (ND),^7^ genetics is another important component, as ND has an average heritability of 0.56.^7^ Of the identified susceptibility genes for ND, the most investigated ones are members of the nicotinic acetylcholine receptor (nAChR) gene family, which encodes 12 subunits (i.e., α2–α7; β2–β4) that are widely expressed in many brain regions.^8–10^ Nicotine, a primary component of tobacco smoke, exerts its biological effects on these nAChRs, where it either stimulates them or inactivates them through desensitization.^11^

Numerous studies using approaches such as genome-wide linkage, candidate gene association, and genome-wide association (GWAS) analysis have greatly advanced our knowledge of the genetic architecture underlying ND.^12^ The most replicated susceptibility loci for ND are nAChR subunit genes in the *CHRNA5/A3/B4* cluster on chromosome 15,^12–24^ *CHRNB3/A6* on chromosome 8,^12,25^ and *CYP2A6/A7* on chromosome 19.^12,14,25^ In addition, a significant association has been reported of variants in the *CHRNA5/A3/B4* cluster with ND and lung cancer.^18^

Although the reported associations of variants in *CHRNA7* with ND have not been replicated consistently in independent samples, pharmacological and molecular studies have strongly implicated α7 as an important target for ND and smoking cessation.^26^ Knockout α7 mice show a greater preference for oral nicotine than do their wild-type counterparts.^27^ In addition, local infusion of a highly selective antagonist (α-conotoxin ArIB) of α7 into the nucleus accumbens (NAc) shell or the anterior cingulate cortex increases nicotine self-administration.^28^

Numerous studies have shown that the β2 subunit is the most abundant of the nAChRs expressed in the brain and has high affinity for nicotine and acetylcholine (Ach). Further, many studies have demonstrated that the β2* (* indicates non-β2 subunits forming a functional nAChR by combining with β2) is required for nicotine reinforcement and reward.^29^ In the studies of therapeutics for smoking cessation, both β2-containing and α7-containing nicotine receptors have proved to be targets to curb tobacco addiction. However, α7 appears to act as modulator of nicotine reinforcement in opposition to α4β2* and α6β2*. Melis et al.^30^ showed a modulatory role for α7 in the ventral tegmental area to reduce β2* activation, especially for α4β2* and α6β2*, by stimulating the intracellular signaling cascades that inhibit β2*. These findings indicate that both α7 and β2* activation play an essential role in ND and smoking cessation.

Exogenous factors such as cigarette smoking can alter DNA methylation either locally or globally.^31^ Persistent exposure to smoke stimulates epigenetic reprogramming at the global level by affecting the methylation of repetitive elements.^32^ Although methylation is also under genetic influence, allele-specific DNA methylation is often correlated in related individuals.^33^ It is thus plausible to infer a correlation between these aberrant CpGs and risk variants for cigarette smoking.

A diversity of susceptibility variants for smoking has been identified, but the mechanisms by which these SNPs contribute to smoking-related traits are generally unclear. Benefits from high-throughput next-generation sequencing and high-density array platforms have allowed researchers to find regulatory variants by mapping expression and methylation quantitative trait loci (meQTLs).^34,35^ This approach provides a better way to reveal the mechanisms of significant variants from association studies. For example, considering the chromosome region of 15q25.1 as a well-established susceptibility region for smoking-related phenotypes, Hancock et al.^36^ assessed the number of methylation loci in the region based on the Illumina HumanMethylation27 array, which led to identification of a novel regulatory SNP, rs11636753, in *CHRNA5* that modulates methylation and expression in ND-relevant brain regions in multiancestry groups. Because the Illumina array contains methylation loci only from promoter regions,^37^ it is necessary to perform a fine-mapping analysis of this region to detect more *cis*-meQTLs.

There were three objectives of this study: (1) to determine which individual SNPs or haplotypes in nAChR subunit genes are associated with ND in a Chinese Han smoker sample, a less commonly investigated population; (2) to detect significant interactive effects among these genes in exerting influence on the etiology of ND; and (3) to link risk variants for ND and differential DNA methylation loci by *cis*-mQTLs analysis to explore the underlying mechanisms involved in ND.

## Materials and methods

### Subjects

This study included 5055 unrelated subjects consisting of both smokers (*N* = 2616) and non-smokers (*N* = 2439), who were recruited from local hospitals in Jincheng and Taiyuan in Shanxi Province of China in 2013. Participants with clinically diagnosed psychiatric disorders such as schizophrenia, Alzheimer’s disease, and major depressive disorder were excluded. Because few Chinese women aged 15 years or older smoke (~2.7%),^5^ only male smokers were included.

A set of structured questionnaires on cigarette smoking; demographic information such as age, education, and annual income; drug or substance use history; and neighborhood environment were administered to each participant by trained researchers. The detailed demographic characteristics of this sample are shown in Table 1. The average age of the smokers was 40.4 ± 9.7 years and that of the non-smokers was 37.0 ± 10.9 years. Written informed consent was provided by each participant after receiving a detailed explanation of the project and process of this study. The study and all the questionnaires used in the study were approved by the Ethics Committee of First Affiliated Hospital of Zhejiang University School of Medicine.

**Table 1:** Characteristics of samples used in the study

Characteristic	Smokers	Non-smokers
Sample size	2616	2439
Age, years (S.D.)	40.4 (9.7)	37.0 (10.9)
FTND in smokers only (S.D.)	5.45 (1.41)	NA
No. of family members smoke (S.D.)	1.9 (0.8)	1.6 (0.7)
Income categories (S.D.)	4.0 (1.2)	4.0 (1.2)

Income categories: (1) less or equal to 10,000 Yuan/years; (2) 10,001–20,000 Yuan/years; (3) 20,001 –30,000 Yuan/years; (4) 30,001–50,000 Yuan/years; (5) over than 50,000 Yuan/years

### Phenotype assessment

Non-smokers were defined as persons who had smoked fewer than 100 cigarettes in their lifetimes. Smoking dependence was assessed by the Fagerström Test for Nicotine Dependence (FTND) measure (0–10 scale).^38,39^ We defined the smokers with an FTND score of ≥6 as heavy smokers (*N* = 1243) and those with an FTND score of <6 as light smokers (*N* = 1373) (Supplementary Table 6).^40^

### Selection of SNPs for genotyping analysis

Peripheral blood was collected from each participant. Genomic DNA was extracted by the Qiagen DNA purification kit according to the manufacturer’s protocol. A nanodrop was utilized to determine the DNA concentration of each sample based on the optical density (OD) at 260 nm, and the DNA quality of each sample was assessed by the OD 260/280 ratio.

Genotyping was performed with the *Taq*man OpenArray Genotyping Platform (Applied Biosystems, Inc.). For each sample, a mixture of 2 μl of DNA (ca. 100 ng) and 2 μl of 2× *Taq*man OpenArray Genotyping Master Mix was added to a 384-well plate. After the plate was sealed and centrifuged briefly, each plate was loaded into the QuantStudio^TM^ 12 K Flex for PCR amplification. The amplified results were reviewed by *Taq*man Genotyper Software (v. 1.3.1) for SNP calling.

The SNPs examined in this study were three in *CHRNB2*, 14 in *CHRNA3*, 10 in *CHRNB4*, 16 in *CHRNA5*, 23 in *CHNRA7*, and 6 in *CHRNA4*. They were all selected with the SNP Browser software from Applied Biosystems by searching the dbSNP database and published papers. However, because of the calling rate of <95%, SNPs rs6495309 in *CHRNA3*, rs11637890 in *CHRNB4*, rs503464 and rs601079 in *CHRNA5*, and rs6494211 in *CHRNA7* were excluded. After those quality control steps, a total of 67 SNPs remained for the association analysis. All of these SNPs have a minor allele frequency (MAF) of >1% and a *p* value of >1 × 10^−4^ in the Hardy–Weinberg equilibrium test.

### Population structure analysis

We used the Structure program (v. 2.3.4)^41^ to assess population stratification for our samples based on the genotyping data for a panel of 30 ancestry informative markers.^42^ Simulation parameters were set to 100,000 burn-ins and 100,000 iterations. To increase the accuracy when inferring admixture and taking account of the samples being recruited from two sites, we set *K* to 2. Our population structure analysis revealed no evidence of population admixture among the samples from the two recruitment sites, so we performed our association analysis on both sets of samples together with the goal of increasing our statistical power (Supplementary Figure 1).

### DNA methylation

DNA methylation data used here were obtained from an ongoing whole-genome bisulfite sequencing project in this laboratory which consisted of 72 subjects selected from the same sample set as used for the abovementioned association analyses on the basis of their age, gender, and smoking status (36 non-smokers; 36 smokers). DNA methylation was identified using the Illumina HiSeq X Ten platform with an average of about 700 million (±75 million) 150-bp paired-end reads per sample. Clean reads were mapped to the hg19 reference genome using Bismark.^43^ We first combined two strands of information of CpG sites and then excluded those CpGs with <5 reads or that overlapped with common variants in the Chinese Han genome (MAF > 0.05). The MAF of each variant was determined by an unpublished Whole Genome Sequencing dataset of a Chinese Han sample (*N* = 1329) in our laboratory.

### Individual SNP-based and haplotype-based association analysis

Individual SNP-based association analysis was performed for both smoking status and FTND score using PLINK (v. 1.07)^44^ under the logistic regression model. Adjusted covariates included age, site (Taiyuan or Jincheng), number of smoking family members, and income. In the haplotype-based association analysis, both pair-wise linkage disequilibrium (LD) and haplotypes were evaluated by Haploview (v. 4.2),^45,46^ and the analysis of those major haplotypes (with a frequency >5%) with each phenotype was performed by HaploStats (v. 1.7.7) in *R* and adjusted for same covariates under the additive model.^47^

### Interaction analysis of variants in *CHRNA3*, *CHRNA4*, *CHRNA5*, *CHRNA7*, *CHRNB2*, and *CHRNB4*

To estimate the epistatic contribution of variants in six nAChR subunit genes to ND, we performed SNP-by-SNP interaction analysis using the GMDR-GPU program developed by our group,^48,49^ performing an exhaustive search of all combinations containing 2–5 SNPs each. The best interaction model was determined according to the following three parameters: (1) the cross-validation consistency (CVC) statistics for the selected variant combination; (2) the predictive accuracy, determined by 10^7^ permutation tests for the selected SNP combinations; and (3) the significant *p* value.

On the basis of chromosomal location and known functional nAChR composition, we separated the genotype file into two: (1) all variants in *CHRNA3, CHRNA5, CHRNB4*, and *CHRNA7*, all of which are located on chromosome 15; and (2) all variants in *CHRNA4, CHRNA5*, and *CHRNB2* because they can form a functional (α4β2)_2_α5 receptor in humans.

### Determination of *cis-*mQTL

Our *cis-*mQTL analysis was restricted to 250 kb upstream and downstream of each SNP. The intervals for adjacent SNPs were combined if they overlapped. Taken together, a total of 8,915 CpG sites were revealed within the intervals of the target genes. Prior to analysis, we removed the low-quality DNA methylation sites with a calling rate of <80%, which left 5580 highly qualified methylated CpGs for *cis*-mQTL analysis.

We choose three significant SNPs for our *cis*-mQTL association analysis according to individual SNP-based analysis, namely rs1948, rs3743075, and rs7178270. They were all intronic polymorphisms associated with the extent of methylation. We used the Matrix eQTL (v. 2.1.1) R package^50^ to test association of the three SNPs, with linear regression under an additive model and adjusted for age, smoking status, and whether the subject was a coal miner. Bonferroni correction was used to define significant associations (i.e., *p* = 0.05/14,161 = 3.5 × 10^−6^), where 14,161 is the total number of associations tested for the abovementioned three significant SNPs (i.e., 4623 for rs3743075, 4743 for r1948, and 4795 for rs7178270).

## Results

### Individual SNP-based association analysis

As shown in Table 2, for smoking status, we found that eight variants in *CHRNA5* and one variant in *CHRNA4* showed significant associations (*p* = 8.0 × 10^−3^ to 4.4 × 10^−2^). However, they were no longer significant after Bonferroni correction for multiple testing.

**Table 2:** SNPs with *p* < 0.05 in individual SNP association on analyses with smoking status and FTND in Chinese samples

Gene	SNP	Function	Major allele	Minor allele	MAF	Smoking status		FTND	
						OR	*p* value	OR	*p* value
*CHRNA3*	rs3743075	Synonymous	C	T	0.47	1.1	0.056	0.8	7.0E−04
	rs3743074	Intronic	A	G	0.46	1.2	0.083	0.8	0.002
	rs6495307	Intronic	C	T	0.22	1.1	0.160	0.9	0.039
	rs3743077	Intronic	C	T	0.23	1.1	0.200	0.8	0.016
	rs660652	3’UTR	G	A	0.22	1.1	0.240	0.9	0.024
	rs2869546	Intronic	T	C	0.23	1.1	0.270	0.8	0.014
*CHRNA5*	rs667282	Intronic	T	C	0.46	1.1	0.008	1.2	0.005
	rs555018	Intronic	A	G	0.21	1.1	0.011	0.8	0.019
	rs621849	Intronic	A	G	0.27	1.1	0.020	0.9	0.024
	rs680244	Intronic	C	T	0.27	1.1	0.021	0.9	0.030
	rs692780	Intronic	G	C	0.22	1.1	0.026	0.8	0.022
	rs588765	Intronic	C	T	0.21	1.1	0.032	0.9	0.025
	rs6495306	Intronic	A	G	0.21	1.1	0.044	0.9	0.039
	rs16969968	Missense	G	A	0.03	1.3	0.042	1.4	0.041
	rs514743	Intronic	A	T	0.22	1.1	0.410	0.9	0.029
	rs647041	Intronic	C	T	0.22	1.1	0.220	0.9	0.023
	rs615470	3’UTR	C	T	0.22	1.1	0.260	0.9	0.028
*CHRNB4*	rs7178270	Intronic	C	G	0.40	1.1	0.110	0.7	2.0E−04
	rs1948	Synonymous	G	A	0.47	1.1	0.120	0.8	6.6E−05
	rs950776	Intronic	T	C	0.17	1.1	0.330	0.8	0.003
*CHRNA4*	rs3827020	Intronic	T	C	0.46	1.0	0.430	1.2	0.015

*Chr* chromosome, *MAF* minor allele frequency, *OR* odds ratio

Members, age, income and site were used as covariates to adjust. See subjects and methods for detail

For the FTND phenotype, we found that many variants showed significant associations prior to correction for multiple testing. However, only rs1948 and rs7178270 in *CHRNB4* and rs3743075 in *CHRNA3* remained significant after Bonferroni correction, with *p* values of 6.6 × 10^−5^, 2.0 × 10^−4^, and 7.0 × 10^−4^, the odds ratio (OR) under the additive model being 0.8, 0.7, and 0.8, respectively (Supplementary Table 1).

### Haplotype-based association analysis

According to the haplotype block definition of Gabriel et al.^46^ there were seven blocks in the *CHRNA5/A3/B4* cluster, four in *CHRNA7*, and one in *CHRNA4* and *CHRNB2* (*D*′ > 0.90) (see Supplementary Figure 2 and Supplementary Figure 3).

As shown in Table 3, for the ND measured by smoking status, we found one haplotype, G-C-C, formed by rs1948, rs7178270, and rs17487223, to be marginally associated with smoking status (Hap-Score 2.13; *p* = 0.0331). For the FTND measure, we detected five major haplotypes in the *CHRNA5/A3/B4* cluster that were significantly associated with ND. Of them, two major haplotypes, C-A and T-G, formed by rs3743075 and rs3743074, showed significant associations with the FTND score (Hap-Score 3.51; *p* = 4.0 × 10^−4^; Hap-Score −3.37; *p* = 8.0 × 10^−4^). Another haplotype, C-T, formed by rs12914385–rs2869546, also showed significance (Hap-Score −3.02; *p* = 2.6 × 10^−3^). Further, we found two other haplotypes, A-G-C and G-C-C, formed by SNPs rs1948, rs7178270, and rs17487223 with a frequency of 0.40 and 0.48, to be significantly associated with FTND (Hap-Score −3.57; *p* = 4.0 × 10^−4^; Hap-Score 3.79; *p* = 2.0 × 10^−4^, respectively). Importantly, the haplotype G-C-C, formed by rs1948, rs7178270, and rs17487223 in *CHRNA5/A3/B4*, was significantly associated with both smoking status and FTND. In addition, we found a haplotype in *CHRNA4* to be marginally associated with FTND (Supplementary Table 2).

**Table 3:** Major haplotypes (frequency > 0.05) associated with smoking status and FTND at *p* < 0.05 in Chinese samples

ND measure	Gene	SNP combination	Haplotype	Hap-Freq	Hap-Score	P-Hap	P-Global
Smoking status	*CHRNA5/A3/B4*	rs1948–rs7178270–rs17487223	G-C-C	0.49	2.13	0.0331	0.1122
		rs684513–rs667282–rs588765–rs6495306–rs17486278–rs680244–rs621849–rs692780–rs951266	C-C-C-A-A-C-A-G-G	0.22	2.66	0.0078	0.0462
FTND	*CHRNA5/A3/B4*	rs684513–rs667282–rs588765–rs6495306–rs17486278–rs680244–rs621849–rs692780–rs951266	C-T-T-G-A-T-G-C-G	0.21	2.27	0.0231	0.0197
		rs647041–rs16969968–rs514743–rs615470–rs660652–rs578776–rs6495307–rs1051730–rs3743077	T-G-T-T-A-G-T-G-T	0.22	2.18	0.0291	0.0258
		rs1948–rs7178270–rs17487223	A-G-C	0.40	−3.57	**0.0004**	0.0007
			G-C-C	0.48	3.79	**0.0002**	
		rs12914385–rs2869546	C-C	0.23	2.47	0.0135	0.0065
			C-T	0.50	−3.02	**0.0026**	
		rs3743075–rs3743074	T-G	0.53	−3.37	**0.0008**	0.0009
			C-A	0.47	3.51	**0.0004**	
	*CHRNA4*	rs2236196–rs3787137–rs3827020–rs1044393–rs3787140	A-G-C-G-T	0.45	−2.14	0.0323	0.2009

Significant association after Bonferroni correction were given in bold

Members, age, income, and miner were used as covariates to adjust

### SNP-by-SNP interaction analysis

Combinations of different nAChR subunits can form various functional nicotinic receptors, which play various physiological roles in both the peripheral and the central nervous systems. To determine whether there exists any epistatic effect among these nAChR subunits, we performed interaction analysis using our own GMDR-GPU program, which revealed two significant interaction models for FTND. The first model consists of SNPs rs16969968 in *CHRNA5* and rs7178270 in *CHRNB4*, with a CVC 7 of 10, prediction accuracy of 55.4%, and empirical *p* value of 8 × 10^−5^ based on 10^7^ permutations. The second significant model consists of SNPs rs904951 and rs7178176 in *CHRNA7*, with a CVC 10 of 10, prediction accuracy of 56.0%, and empirical *p* value of 5.8 × 10^−6^ from 10^7^ permutations (Supplementary Table S3). Although we also did interaction analysis on SNPs from *CHRNA3*, *CHRNB4*, and *CHRNA5* with *CHRNA7*, we did not find any significant interaction for variants in *CHRNA7* with those from the other nAChR subunit genes.

For smoking status, we detected a best interaction model, which consists of rs16969968 in *CHRNA5*, rs4845378 and rs3811450 in *CHRNB2*, and rs3787137 and rs1044393 in *CHRNA4*. This model has a CVC 7 of 10, prediction accuracy of 52.3%, and empirical *p* value of 0.012 (Supplementary Table S3).

### Relations between genotype and methylation status

**Fig. 1:**
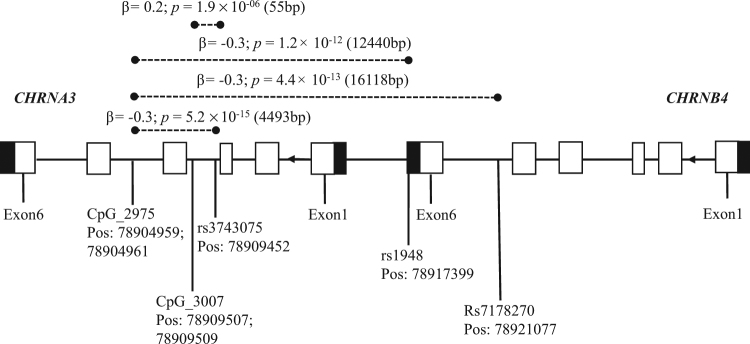
Schematic diagram of mQTL analysis and position of investigated SNPs. The open rectangles represent the exons, black rectangles are untranslated regions, and solid lines indicate introns according to NCBI and UCSC Browsers. Dashed lines refer to the significant SNP–CpG pair. Pos: genome position of SNP and CpG site

**Fig. 2:**
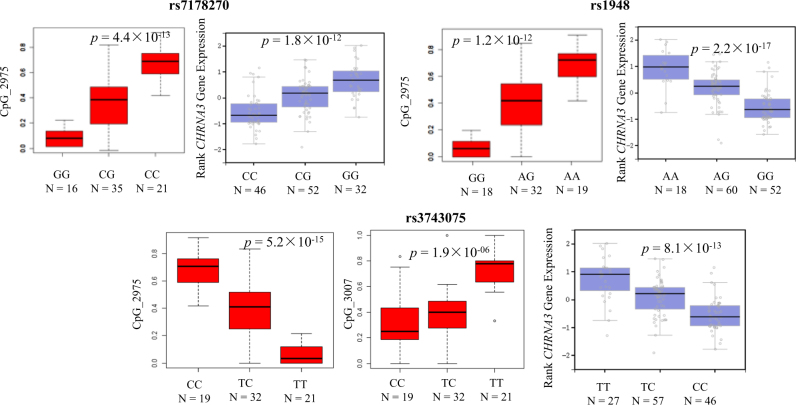
Association between genotype data of risk variants and extent of methylation in blood samples of 72 participants of Chinese Han origin tested by *cis*-mQTLs. Distribution of the values at the methylation sites is presented for individuals carrying zero, one, and two minor alleles. Only the most significant *cis*-mQTLs are shown for each variant. The *cis*-eQTL analysis associated three variants with *CHRNA3* expression in human NAc basal ganglia. The *cis*-eQTL figures were downloaded from GTEX PORTAL (https://gtexportal.org/home/)

All association analysis results of top SNPs with *cis*-mQTLs are shown in Supplementary Table 4 and Supplementary Figure 4 (*p* < 5.0 × 10^−4^). Together, we detected four significant associations for SNP–CpG pairs (Fig. 1). These pairs were formed by three SNPs significant for ND (rs3743075 in *CHRNA3* and rs1948 and rs7178270 in *CHRNB4*) and two distinct CpG sites (CpG_2975, CpG_3007). Significant *cis-*mQTL analysis results and the corresponding *p* values, with a range of 5.2 × 10^−15^ to 1.9 × 10^−06^ (Fig. 2).

Given that the two highly methylated CpG sites are both located in *CHRNA3*, our association analyses of the three SNPs with RNA expression were conducted only for *CHRNA3*. According to the *cis*-eQTL association data from GTEX PORTAL (https://gtexportal.org/home/), we found that the three SNPs linked to ND (i.e., SNPs rs1948, rs7178270, and rs3743075) correlated significantly with two methylation sites that showed allele-specific mRNA expression of *CHRNA3* and *CHRNA5* in several ND-related brain regions of human post-mortem tissue (Supplementary Table 5), with *p* values ranging from 3.2 × 10^−6^ to 4.0 × 10^−15^.

In addition, we performed cis-mQTL analysis by adjusting with FTND score and age. Because the FTND score is a ND measure for smokers, we only included smokers in this analysis. We obtained consistent results (Supplementary Table 7). The significant CpG–SNP pairs were still formed by CpG_2975 with rs3743075, rs1948 and rs7178270, respectively (*p* = 2.2 × 10^−6^; *p* = 1.8 × 10^−5^; *p* = 2.4 × 10^−5^). These results further support that these three variants are risk variants that influence the extent of methylation in smokers.

## Discussion

To our knowledge, this is the first study exploring the underlying mechanisms of ND using multiple approaches of genetic associations, interaction, and *cis-*mQTLs in a Chinese Han population. Our results revealed that three SNPs and five major haplotypes in *CHRNA5/A3/B4* were significantly associated with the FTND score. We also demonstrated that the significant ND-associated variants (rs1948, rs7178270, and rs3743075) are novel *cis*-meQTLs influencing both the extent of methylation and mRNA expression of *CHRNA3*. In addition, through SNP-by-SNP interaction study, we found several SNPs in *CHRNA5/B4*, *CHRNA5/A4/B2*, and *CHRNA7* that interactively confer susceptibility to ND in our Han sample.

Two haplotypes formed by rs3743074 and rs3743075 were significantly associated with the FTND score. Of them, rs3743075 was significantly and rs3743074 was marginal associated with the score. Further, we showed that the minor allele of rs3743075 in *CHRNA3* was significantly associated with the extent of methylation of CpG_2975 and CpG_3007 (*p* = 5.2 × 10^−15^ and *p* = 1.9 × 10^−6^, respectively) and marginally associated with CpG_3006 (*p* = 4.7 × 10^−4^). In concert with these findings, Hancock et al.^36^ reported that rs3743075 was negatively correlated with a methylation locus of cg22670733 in adult brain (*p* = 7.0 × 10^−6^). According to the annotation by GTEX PORTAL (https://gtexportal.org/home/), rs3743075 has strong evidence of affecting expression of *CHRNA3* in the brain nucleus accumnems (NAc) (*p* = 4.9 × 10^−11^)^51^ and of *CHRNA5* in the NAc, anterior cingulate cortex (BA 24), frontal cortex (BA 9), hippocampus and whole blood (*p* = 2.3 × 10^−10^; *p* = 4.9 × 10^−8^; *p* = 3.0 × 10^−10^; *p* = 1.6 × 10^−5^)^52^ as a *cis*-eQTL in humans. By using the Web-based tool SWISS-PROT (http://www.uniprot.org/), we found that rs3743075 represents a part of the conserved transcription factor-binding site for interferon regulatory factor 7 (*IRF-7*) in humans. Previously, we showed that the expression of *IRF-7* mRNA was significantly suppressed by nicotine treatment in mouse RAW264.7 macrophages.^53^ Thus, it is highly likely that rs3743075 is a functional variant regulating the expression of *CHRNA3* by altering the methylation contribution. In light of previous evidence demonstrating that smoking-associated abnormal methylation loci might convey a risk for lung cancer,^54,55^ this may indicate that rs3743075 is a biomarker for lung cancer.

SNPs rs1948 and rs7178270 in *CHRNB4* showed the strongest association with the FTND score in our Chinese Han sample. In addition, we found that two haplotypes, A-G-C and G-C-C (formed by rs1948, rs7178270, and rs17487223), showed significant associations with the FTND score. Moreover, we demonstrated that the minor allele of rs1948 and rs7178270 significantly reduced the methylation of CpG_2975 (*p* = 4.4 × 10^−13^ and *p* = 1.2 × 10^−12^, respectively). Interestingly, these two SNPs increased the expression of *CHRNA3* in the NAc.^51^ These results indicate that SNPs rs1948 and rs7178270 confer susceptibility to ND by suppressing the methylation that leads to increased expression of *CHRNA3*, which is consistent with previous documentation performed with Dutch persons born in The Netherlands.^56^ For *CHRNA5*, there were eight SNPs showing marginal associations with both smoking status and FTND (*p* < 0.05). Among them, two SNPs, rs16969968 and rs667282 in *CHRNA5*, have been widely associated with ND-related phenotypes and lung cancer in subjects of multiancestry, such as Europeans, Africans, and Asian.^12–14,17,19,22,24,57,58^ In addition, there exists one haplotype consisting of nine variants in the *CHRNA5/A3/B4* cluster that is associated marginally with the FTND score and two haplotypes, formed by another nine SNPs in the *CHRNA5/A3/B4* cluster, that are associated marginally with both the FTND and smoking status.

We performed gene–gene interaction analysis of all selected SNPs within *CHRNA5/A3/B4* and *CHRNA7*. We detected the best interaction model between rs7178270 in *CHRNB4* and rs16969968 in *CHRNA5* (*p* = 8.0 × 10^−5^) with FTND, providing genetics-based evidence supporting the view that *CHRNB4* interacts with *CHRNA5* in contributing to ND. Previous molecular studies found that in mice, medial habenula overexpression of *Chrnb4* strongly increases the aversive effect of nicotine, which can be reversed by lentiviral-mediated expression of *Chrna5* D398N.^59^ Moreover, we observed that the interaction model of (α4β2)_2_α5 grouped by five SNPs (rs4845378, rs3811450, rs3787137, rs1044393, and rs16969968) was significantly associated with smoking status (*p* = 0.012). This interactive model was compatible with biological evidence that (α4β2)_2_α5 carrying the risk allele of rs16969968 has a twofold lower maximum response with a nicotinic agonist compared with the α5 carrying a protective allele.^60^ This is the first evidence of an epistatic effect of this gene cluster discovered via genetic methods in the Chinese Han population.

To date, there are far fewer loci in *CHRNA7* reported to be significantly associated with smoking-related phenotypes, in contrast to the numerous reports that the gene is essential in ND in pharmacological studies. In the present work, we did not detect any significant association by either individual SNP-based or haplotype-based association analysis. One possible explanation for the inconsistent observations is that the variants in *CHRNA7* contribute to ND risk through SNP-by-SNP interactions. Concordant with this assumption, we found a significant interaction model formed by rs904951 and rs7178176 associated with FTND. rs7178176 was previously reported in association with an increasing probability of dizziness at first smoke inhalation by adolescents in a Canada sample with a mixed ethnical origins.^61^ For the first time, we provide evidence that an epistatic effect of *CHRNA7* is implicated in ND, which is in accord with the biological fact that (α7)_5_ forms a homomeric pentamer in humans.

There are a few limitations of this study. First, the number of subjects in the methylation cohort might be too small to detect weak signals of risk polymorphisms and mapping *trans*-mQTLs. Considering that *trans*-meQTL is less common and contributes only minor effects to phenotypes,^62^ we concentrated our analysis on identification of *cis*-meQTLs. In spite of the small samples, because we adopted stringent criteria to select high-quality CpG sites, we believe that our *cis*-meQTL analysis results are trustworthy and deserve to be replicated by others if possible. Second, we chose 67 variants within the six genes to perform association analysis for our sample, and the majority of them are common SNPs. Thus, we could not assess the effects of rare variants, which have been thought to exert more influence on traits of interest. Further next-generation sequencing-based studies are warranted to identify more rare variants within these regions for ND in the Chinese Han population.

To sum up, this is the first study to demonstrate the significant effects of *CHRNA3–CHRNB4* variants on ND in the Chinese Han population. Having succeeded in performing integrative data analysis for genetic polymorphisms and DNA methylation, we relate SNP-based association, *cis*-mQTL analysis, and mRNA expression using public data to explore the biological mechanisms of ND. Further, we provide novel evidence of a significant genetic interactive model for *CHRNA7* in affecting ND, which extends our knowledge of the potential biological mechanism for this gene’s actions affecting ND. A complete understanding of the genetic variants will help us find pharmacologic targets to account for the addictive properties of nicotine in Chinese smokers.

## Electronic supplementary material

Supplementary Figures

Supplementary Tables
