# Supplementary material for: Association and cis-mQTL analysis of variants in CHRNA3-A5, CHRNA7, CHRNB2, and CHRNB4 in relation to nicotine dependence in a Chinese Han population
Source: Transl Psychiatry. 2018 Apr 18;8:83. doi: 10.1038/s41398-018-0130-x (PMC5904126; doi:10.1038/s41398-018-0130-x)
Supplement: Supplementary file 1 — Supplementary Figures [file 41398_2018_130_MOESM1_ESM.pdf]

Supplementary Data

Figure S1. Clustering results of AIMs of Chinese Han population. Each color represents collection site for a sample. Red: Taiyuan; Green: Jincheng.

Figure S2. The LD structures for *CHRNA5/A3/B4* in the Chinese sample were drawn with Haploview (v. 4.2),[45] and haplotype blocks were defined according to Gabriel *et al*.[46] The arrow above the figure represents the gene transcription direction from 5′ to 3′.

Figure S3. The LD structures for CHRNA7, CHRNA4, and CHRNB2 in the Chinese sample were drawn by Haploview (v. 4.2),45 and haplotype blocks were defined according to Gabriel et al.46 The arrow above the figure represents the gene transcription direction from 5′ to 3′.

Figure S4. Associations between genotype data of risk-nominating variants and extent of methylation in blood of 72 Chinese Han participants tested for *cis*-mQTLs. Distribution of the values at the methylation sites is presented for individuals carrying zero, one, and two minor alleles. Only the marginally significant *cis*-mQTLs are shown for each variant ($p < 4.7 \times 10^{-4}$).

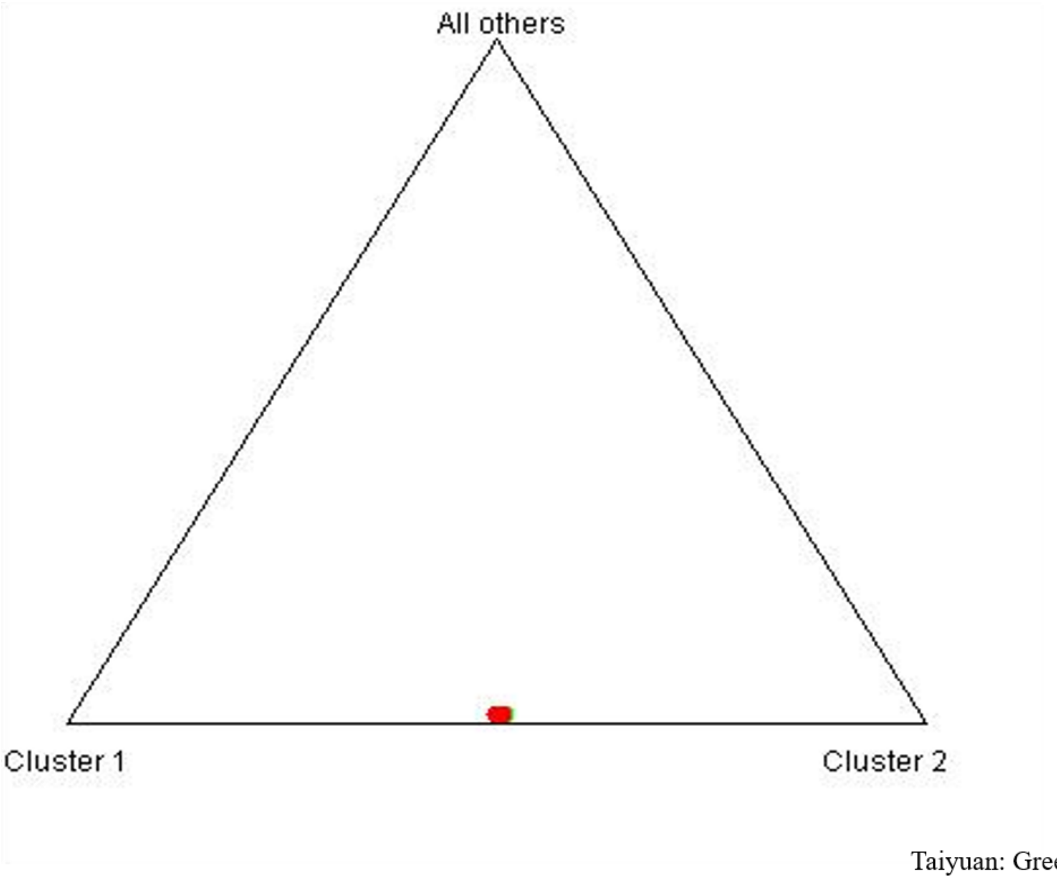

Taiyuan: Green
Jincheng: Red

Supplementary Figure S1

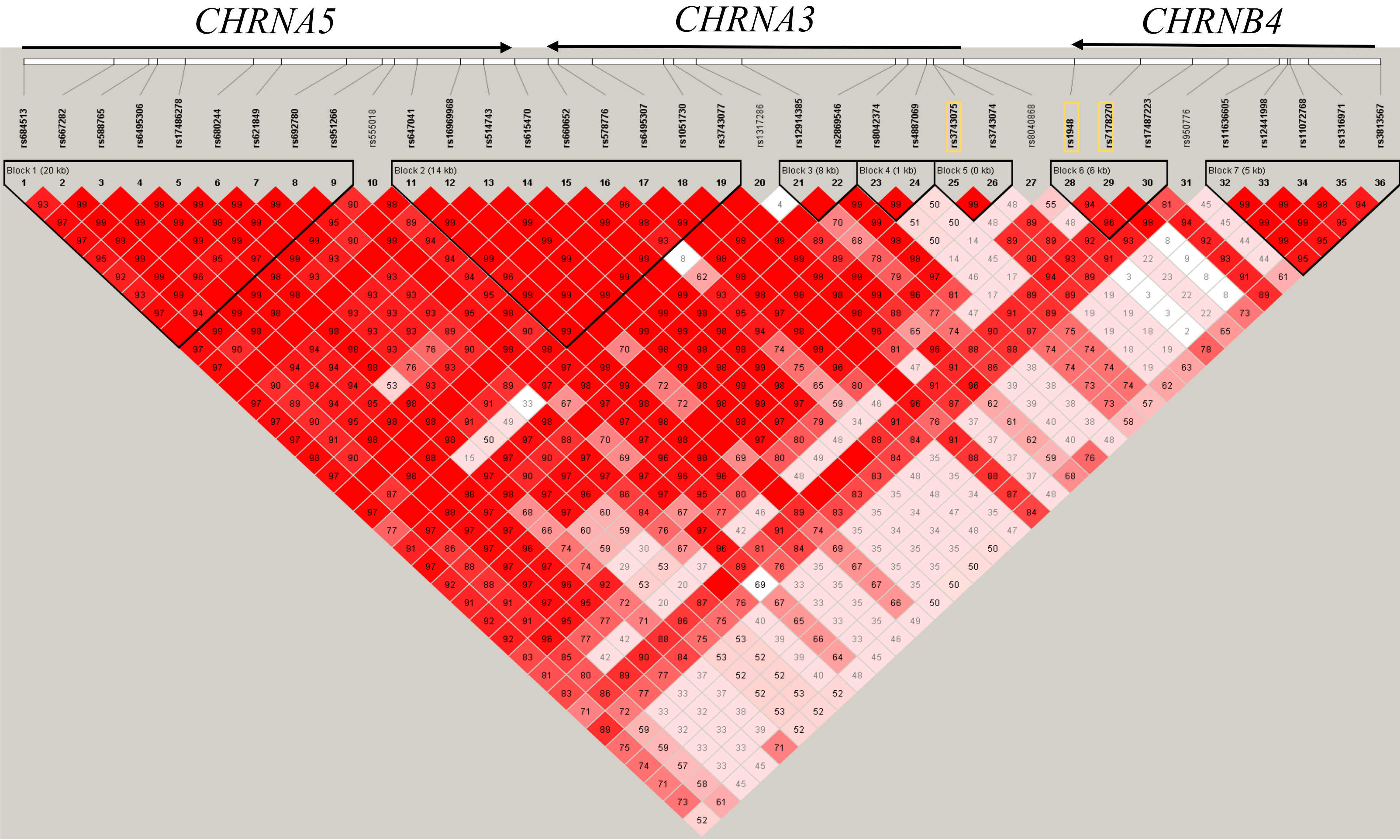

Supplementary Figure S2

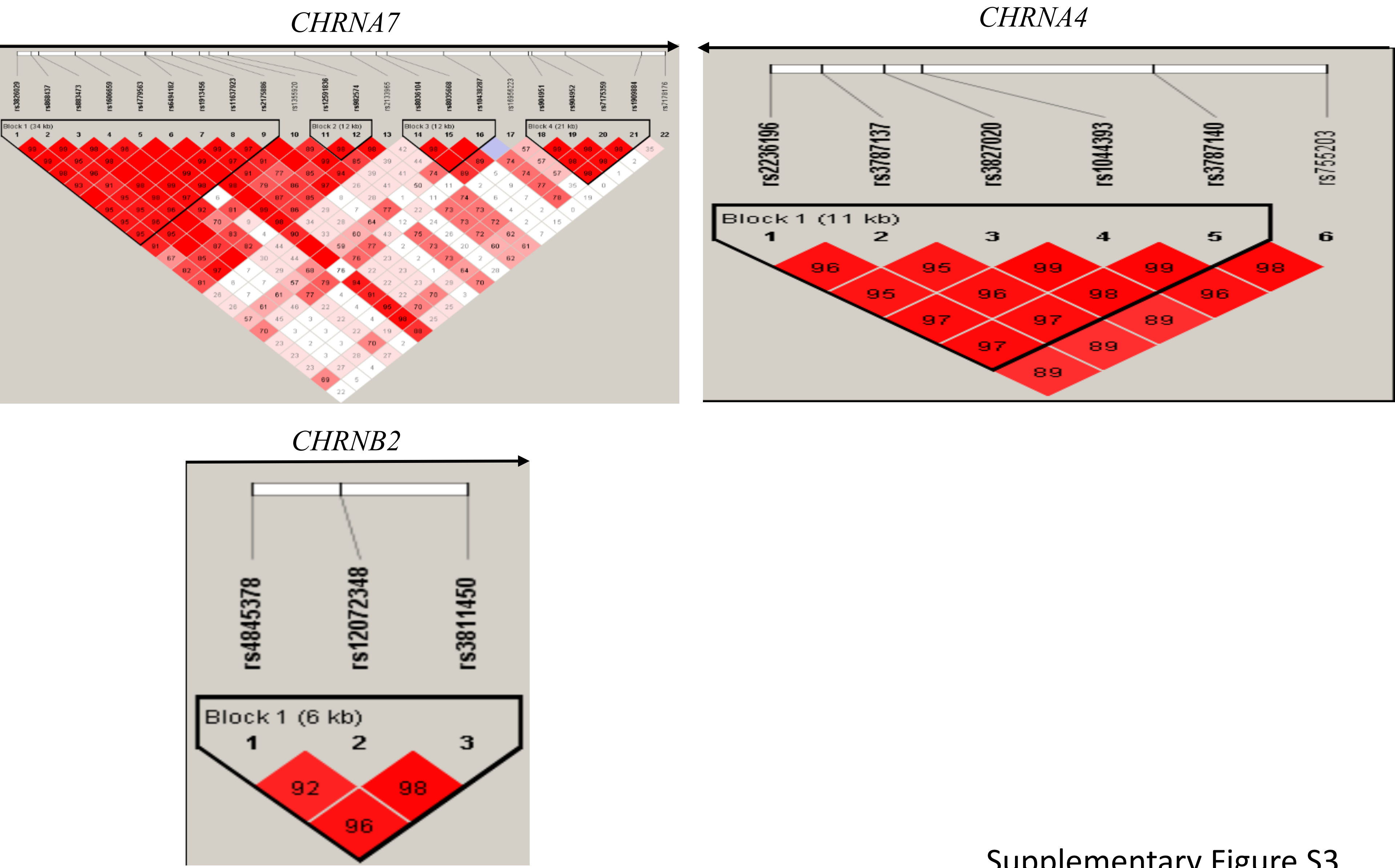

Supplementary Figure S3

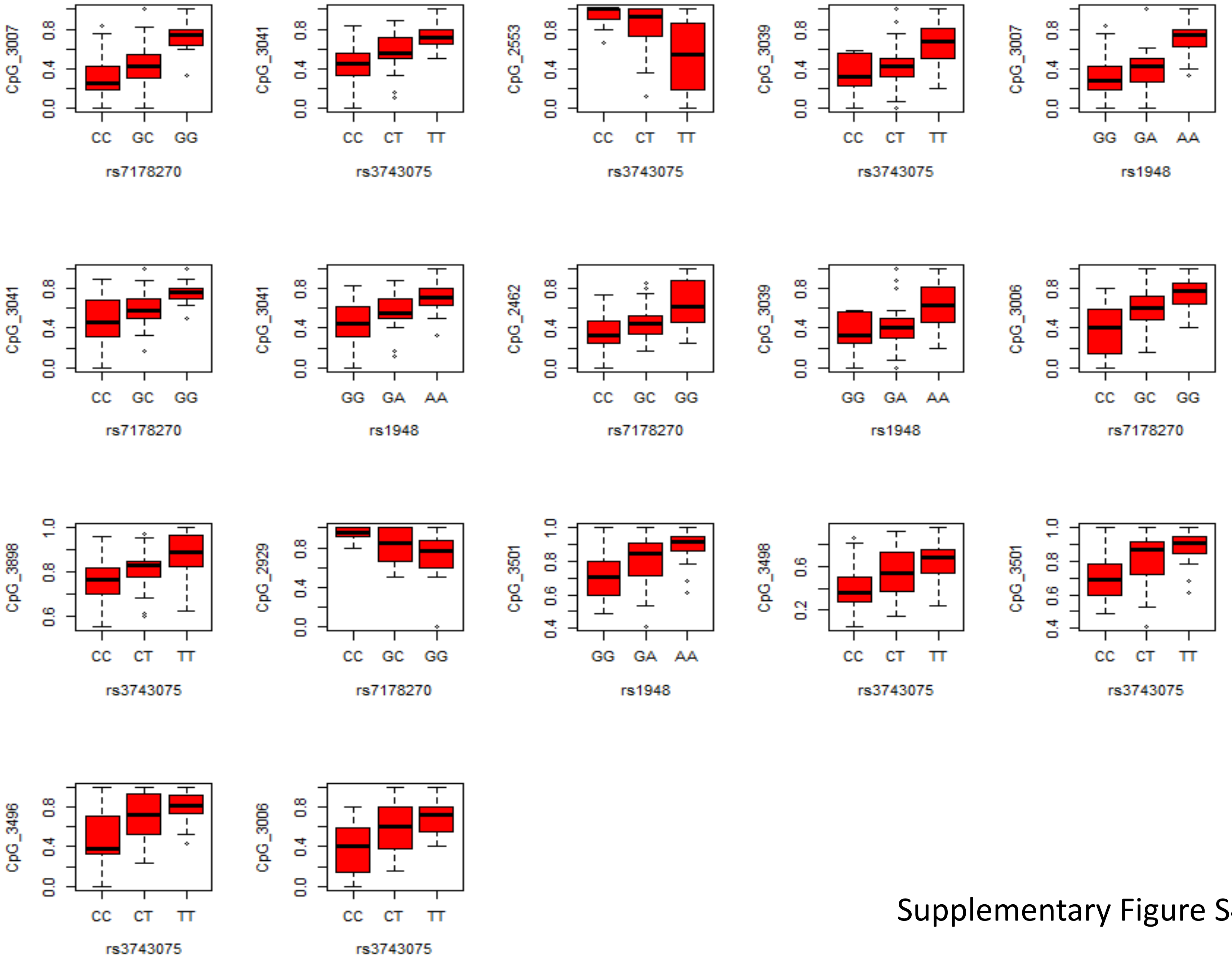

Supplementary Figure S4
