# Supplementary material for: Association and cis-mQTL analysis of variants in CHRNA3-A5, CHRNA7, CHRNB2, and CHRNB4 in relation to nicotine dependence in a Chinese Han population
Source: Transl Psychiatry. 2018 Apr 18;8:83. doi: 10.1038/s41398-018-0130-x (PMC5904126; doi:10.1038/s41398-018-0130-x)
Supplement: Supplementary file 2 — Supplementary Tables [file 41398_2018_130_MOESM2_ESM.pdf]

Table S1 Association *p* values of each SNP in individual with Smoking Status and FTND in Chinese samples

Chr	SNP	GENE	Major Allele	Minor Allele	Function	MAF	Smoking Status		FTND	
							OR	*p* value	OR	*p* value
1	rs12072348	*CHRNB2*	A	C	intronic	0.18	0.90	0.370	0.90	0.130
	rs4845378		G	T	intronic	0.17	1.00	0.690	0.90	0.160
	rs3811450		C	T	3'UTR	0.15	1.00	0.490	1.00	0.600
15	rs3743075	*CHRNA3*	C	T	synonymous	0.47	1.10	0.056	0.80	7.0E-04
	rs3743074		A	G	intronic	0.46	1.20	0.083	0.80	0.002
	rs2869546		T	C	intronic	0.23	1.10	0.270	0.80	0.014
	rs3743077		C	T	intronic	0.23	1.10	0.200	0.80	0.016
	rs660652		G	A	3'UTR	0.22	1.10	0.240	0.90	0.024
	rs6495307		C	T	intronic	0.22	1.10	0.160	0.90	0.039
	rs8042374		G	A	intronic	0.31	1.00	0.260	0.90	0.076
	rs4887069		G	A	intronic	0.31	1.10	0.100	0.90	0.082
	rs1051730		G	A	synonymous	0.03	1.10	0.410	1.40	0.094
	rs578776		A	G	3'UTR	0.25	1.10	0.078	0.90	0.160
	rs1317286		A	G	intronic	0.09	1.10	0.500	1.10	0.220
	rs8040868		T	C	synonymous	0.32	1.10	0.230	0.90	0.300
	rs12914385		C	T	intronic	0.26	1.00	0.350	0.90	0.300
	rs667282	*CHRNA5*	T	C	intronic	0.46	1.10	0.008	1.20	0.005
	rs555018		A	G	intronic	0.21	1.10	0.011	0.80	0.019
	rs692780		G	C	intronic	0.22	1.10	0.026	0.80	0.022
	rs647041		C	T	intronic	0.22	1.10	0.220	0.90	0.023
	rs621849		A	G	intronic	0.27	1.10	0.020	0.90	0.024

	rs588765		C	T	intronic	0.21	1.10	0.032	0.90	0.025
	rs615470		C	T	3’UTR	0.22	1.10	0.260	0.90	0.028
	rs514743		A	T	intronic	0.22	1.10	0.210	0.90	0.029
	rs680244		C	T	intronic	0.27	1.10	0.021	0.90	0.030
	rs6495306		A	G	intronic	0.21	1.10	0.044	0.90	0.039
	rs16969968		G	A	missense	0.03	1.30	0.042	1.40	0.041
	rs951266		G	A	intronic	0.03	1.10	0.460	1.30	0.067
	rs684513		C	G	intronic	0.24	1.00	0.860	1.10	0.120
	rs17486278		A	C	intronic	0.27	1.00	0.490	0.90	0.290
	rs1948	*CHRNB4*	G	A	synonymous	0.47	1.10	0.120	0.80	6.6E-05
	rs7178270		C	G	intronic	0.40	1.10	0.110	0.70	2.0E-04
	rs950776		T	C	intronic	0.17	1.10	0.330	0.80	0.003
	rs3813567		A	G	5' near gene	0.42	1.00	0.230	1.10	0.077
	rs17487223		C	T	intronic	0.03	1.10	0.350	1.40	0.085
	rs11072768		T	G	intronic	0.22	1.00	0.370	0.90	0.230
	rs11636605		A	G	intronic	0.22	1.10	0.250	0.90	0.290
	rs12441998		G	A	intronic	0.22	1.00	0.480	0.90	0.300
	rs1316971		A	G	intronic	0.22	1.00	0.810	0.90	0.340
	rs1913456	*CHRNA7*	T	A	intronic	0.38	1.00	0.490	1.10	0.310
	rs16956223		G	A	intronic	0.02	0.90	0.670	1.20	0.340
	rs1606659		A	G	intronic	0.37	1.00	0.310	1.10	0.420
	rs3826029		T	C	5' near gene	0.38	1.00	0.300	1.00	0.440
	rs11637923		T	C	intronic	0.40	1.00	0.560	1.00	0.490
	rs8035668		A	G	intronic	0.06	1.00	1.000	0.90	0.510
	rs4779563		T	C	intronic	0.27	1.00	0.590	1.00	0.550

	rs904951		G	A	intronic	0.29	1.00	0.730	1.00	0.580
	rs6494182		G	A	intronic	0.12	1.00	0.690	1.10	0.590
	rs10438287		A	G	intronic	0.05	1.00	0.910	0.90	0.590
	rs904952		T	C	intronic	0.29	1.00	0.950	1.00	0.590
	rs8036104		A	C	intronic	0.06	1.00	0.970	0.90	0.630
	rs7178176		C	T	intronic	0.09	0.90	0.130	1.00	0.680
	rs982574		C	G	intronic	0.19	1.00	0.810	1.00	0.730
	rs12591836		T	G	intronic	0.07	0.90	0.230	1.00	0.760
	rs1355920		A	G	intronic	0.18	1.00	0.910	1.00	0.760
	rs1909884		G	A	intronic	0.26	1.00	0.360	1.00	0.790
	rs7175359		C	T	intronic	0.29	1.00	0.630	1.00	0.820
	rs2133965		G	A	intronic	0.18	1.00	0.890	1.00	0.840
	rs868437		C	T	intronic	0.20	1.10	0.240	1.00	0.860
	rs883473		A	G	intronic	0.20	1.10	0.320	1.00	0.910
	rs2175886		C	T	intronic	0.20	1.10	0.230	1.00	0.940
20	rs3827020	*CHRNA4*	T	C	intronic	0.46	1.00	0.430	1.20	0.015
	rs3787137		G	A	intronic	0.36	1.10	0.240	0.90	0.140
	rs755203		G	A	intronic	0.35	1.10	0.240	0.90	0.320
	rs3787140		T	C	intronic	0.17	0.90	0.082	0.90	0.440
	rs2236196		A	G	3’UTR	0.18	0.90	0.034	0.90	0.450
	rs1044393		G	A	synonymous	0.17	0.90	0.086	0.90	0.450

Notes: 1) Chr: Chromosome; MAF: Minor Allele Frequency; OR: Odds Ratio. 2) Members, Age, Income and Site were used as covariates to adjust all statistical model. See subjects and methods for details.

Table S2. Major Haplotypes (frequency >0.05) Associated with Smoking Status and FTND in Chinese samples

			Smoking Status				FTND			
Gene	SNP Combination	Haplotype	Hap-Freq	Hap-Score	P-Hap	P-Global	Hap-Freq	Hap-Score	P-Hap	P-Global
CHRNB2	rs4845378-rs12072348-rs3811450	T-C-C	0.16	0.10	0.9213	0.4804	0.16	1.07	0.2829	0.6587
		G-A-T	0.15	0.18	0.8546		0.15	0.21	0.8350	
		G-A-C	0.66	0.34	0.7365		0.66	-1.13	0.2587	
CHRNA4	rs2236196-rs3787137-rs3827020-rs1044393-rs3787140	G-G-T-A-C	0.16	-1.75	0.0806	0.2067	0.17	0.71	0.4758	0.2009
		A-G-C-G-T	0.45	0.60	0.5513		0.45	-2.14	0.0323	
		A-A-T-G-T	0.35	1.14	0.2525		0.34	1.54	0.1245	
CHRNA7	rs3826029-rs868437-rs883473-rs1606659-rs4779563-rs6494182-rs1913456-rs11637923-rs2175886-	C-T-G-G-C-G-A-C-T	0.19	-1.26	0.2091	0.2303	0.19	0.03	0.9760	0.2303
		C-C-A-G-T-A-A-C-C	0.11	-0.66	0.5087		0.12	-0.36	0.7188	
		T-C-A-A-T-G-T-T-C	0.58	0.15	0.8809		0.58	0.55	0.5795	
		C-C-A-G-C-G-A-C-C	0.06	0.76	0.4484		0.06	-0.89	0.3749	

	rs12591836-rs982574	T-C	0.81	-0.49	0.6220	0.4614	0.81	0.37	0.7112	0.4614
		T-G	0.12	-0.39	0.6943		0.12	-0.22	0.8251	
		G-G	0.06	1.18	0.2393		0.06	-0.35	0.7244	
	rs8036104-rs8035668-rs10438287	A-A-A	0.88	-0.08	0.9339	0.8381	0.88	-0.75	0.4540	0.7280
		C-G-A	0.06	0.14	0.8912		0.06	0.73	0.4637	
		A-A-G	0.05	0.17	0.8636		NA	NA	NA	
	rs904951-rs904952-rs7175359-rs1909884	G-T-C-A	0.26	-0.94	0.3467	0.8232	0.27	-0.01	0.9897	0.9008
		A-C-T-G	0.29	0.25	0.8059		0.28	-0.27	0.7856	
		G-T-C-G	0.44	0.57	0.5665		0.44	0.39	0.6951	
CHRNA3/B4/A5	rs684513-rs667282-rs588765-rs6495306-rs17486278-rs680244-rs621849-rs692780-rs951266	C-T-T-G-A-T-G-C-G	0.20	-1.68	0.0936	0.0462	0.21	2.27	0.0231	0.0197
		C-T-C-A-C-C-A-G-G	0.23	-0.48	0.6341		0.24	1.78	0.0753	
		G-C-C-A-A-C-A-G-G	0.23	0.52	0.6012		0.23	-1.71	0.0873	
		C-C-C-A-A-C-A-G-G	0.22	2.66	0.0078		0.21	1.84	0.0651	
	rs647041-rs16969968-rs514743-rs615470-rs660652-rs578776-rs6495307-rs1051730-rs3743077	T-G-T-T-A-G-T-G-T	0.21	-1.25	0.2097	0.4184	0.22	2.18	0.0291	0.0258
		C-G-A-C-	0.74	1.30	0.1938		0.73	-1.18	0.2367	

		G-A-C-G-C								
	rs12914385-rs2869546	T-T	0.26	-1.05	0.2929	0.3311	0.27	1.04	0.2969	0.0065
		C-C	0.23	-0.91	0.3645		0.23	2.47	0.0135	
		C-T	0.51	1.67	0.0945		0.50	-3.02	**0.0026**	
	rs8042374-rs4887069	A-A	0.31	-1.30	0.1937	0.1989	0.32	1.60	0.1106	0.1033
		G-G	0.69	1.45	0.1479		0.68	-1.78	0.0758	
	rs3743075-rs3743074	T-G	0.46	-1.69	0.0909	0.2285	0.53	-3.37	**0.0008**	0.0009
		C-A`	0.53	1.71	0.0870		0.47	3.51	**0.0004**	
	rs1948-rs7178270-rs17487223	A-G-C	0.39	-1.27	0.2026	0.1122	0.40	-3.57	**0.0004**	0.0007
		A-C-C	0.07	-0.55	0.5836		0.08	1.15	0.2487	
		G-C-C	0.49	2.13	0.0331		0.48	3.79	**0.0002**	
	rs11636605-rs12441998-rs11072768-rs1316971-rs3813567	G-A-G-G-A	0.21	-0.80	0.4221	0.5647	0.22	1.22	0.2244	0.4189
		A-G-T-A-A	0.36	-0.72	0.4717		0.36	0.56	0.5761	
		A-G-T-A-G	0.42	1.25	0.2113		0.41	-1.59	0.1120	

Notes: 1) Significant given in bold; 2) Members, Age, Income and Miner were used as covariates to adjust all statistical model.

Supplementary Table S3. Summary of detected interaction models in Chinese sample

ND Measure	SNP Combinations	CVC	Prediction Accuracy	*p* value
Smoking Status	*CHRNB2*: rs4845378, rs3811450<br>*CHRNA4*: rs3787137, rs1044393<br>*CHRNA5*: rs16969968	7/10	52.3%	0.012
FTND	*CHRNA5*: rs16969968<br>*CHRNB4:* rs7178270	7/10	55.4%	8.0E-05
	CHRNA7: rs904951, rs7178176	10/10	56.0%	5.8E-06

Table S4. SNPs associated with methylation level at nearby CpGs (p < 5.0E-04) in blood of Chinese Sample (N = 72)

Chr	CpGs	CpGs Position		Associated Variant	SNP Position	Distance to SNP (bp)	Beta	*p* value
		Start	End					
Chr15	CpG_2975	78904959	78904961	rs3743075	78909452	-4493	-0.3	**5.2E-15**
	CpG_2975	78904959	78904961	rs7178270	78921077	-16118	-0.3	**4.4E-13**
	CpG_2975	78904959	78904961	rs1948	78917399	-12440	-0.3	**1.2E-12**
	CpG_3007	78909507	78909509	rs3743075	78909452	55	0.2	**1.9E-06**
	CpG_3007	78909507	78909509	rs7178270	78921077	-11570	0.2	4.3E-06
	CpG_3041	78912132	78912134	rs3743075	78909452	2680	0.1	6.3E-06
	CpG_2553	78862844	78862846	rs3743075	78909452	-46608	-0.2	8.5E-06
	CpG_3039	78912078	78912080	rs3743075	78909452	2626	0.2	9.6E-06
	CpG_3007	78909507	78909509	rs1948	78917399	-7892	0.2	1.1E-05
	CpG_3041	78912132	78912134	rs7178270	78921077	-8945	-0.1	8.0E-05
	CpG_3041	78912132	78912134	rs1948	78917399	-5267	-0.1	1.2E-04
	CpG_2462	78857085	78857087	rs7178270	78921077	-63992	0.1	1.4E-04
	CpG_3039	78912078	78912080	rs1948	78917399	-5321	0.1	1.6E-04
	CpG_3006	78909477	78909479	rs7178270	78921077	-11600	0.1	1.7E-04
	CpG_3898	79006008	79006010	rs3743075	78909452	96556	0.1	1.7E-04
	CpG_2929	78899002	78899004	rs7178270	78921077	-22075	-0.1	1.7E-04
	CpG_3501	78958685	78958687	rs1948	78917399	41286	0.1	2.1E-04
	CpG_3498	78958523	78958525	rs3743075	78909452	49071	0.1	3.0E-04
	CpG_3501	78958685	78958687	rs3743075	78909452	49233	0.1	3.6E-04
	CpG_3496	78958460	78958462	rs3743075	78909452	49008	0.1	4.0E-04
	CpG_3006	78909477	78909479	rs3743075	78909452	25	0.1	4.7E-04

Notes: 1) bp: base pair; Chr: Chromosome; Variant Position: SNPs position in chromosome; Distance: the distance from associated SNP to CpG site; SNP: single-nucleotide polymorphisms; Unadj: Unadjusted; 2) Age and Smoking Status as covariates to adjust the methylation level; 3) Significant association were given in bold.

Table S5. SNPs associated with *CHRNA3* and *CHRNA5* expression levels in several different brain regions of human

Gene	Tissue	Associated SNPs	Beta value	*p* value
CHRNA3	Brain Nucleus Accumbens (Basal Ganglia)	rs1948	-0.81	5.9E-15
		rs7178270	0.67	1.8E-12
		rs3743075	-0.71	8.1E-13
CHRNA5	Brain Nucleus Accumbens (Basal Ganglia)	rs1948	-0.65	2.3E-10
		rs7178270	0.69	4.0E-15
		rs3743075	-0.70	1.1E-13
	Anterior Cingulate Cortex (BA 24)	rs1948	-0.69	4.9E-08
		rs3743075	-0.75	1.8E-10
		rs7178270	0.74	4.5E-12
	Frontal Cortex (BA 9)	rs1948	-0.69	3.0E-10
		rs3743075	-0.80	4.3E-10
		rs7178270	0.66	1.7E-10
	Hippocampus	rs1948	-0.60	3.2E-06
		rs3743075	-0.68	2.2E-09
		rs7178270	0.58	5.2E-08

Notes: These data were download from GTEX PORTAL (https://gtexportal.org/home/).

Table S6. The distribution of FTND score with smokers

Characteristic of FTND		
	FTND Score	Sample Size
Light smoker (N = 1,373)	0	1
	1	1
	2	8
	3	241
	4	337
	5	785
Heavy smoker (N = 1,243)	6	751
	7	290
	8	130
	9	62
	10	10

Table S7. SNPs associated with methylation level at nearby CpGs ($p < 5.0E-04$) in blood of smokers of Chinese Sample (N = 36)

Chr	CpGs	CpGs Position		Associated Variant	SNP Position	Distance to SNP (bp)	Beta	*p* value
		Start	End					
Chr15	CPG_2975	78904959	78904961	rs3743075	78909452	-4493	-0.2	2.2E-06
	CPG_2975	78904959	78904961	rs1948	78917399	-12440	-0.2	1.8E-05
	CPG_2975	78904959	78904961	rs7178270	78921077	-16118	-0.2	2.0E-05
	CPG_2685	78875861	78875863	rs1948	78917399	-41538	-0.1	2.9E-05
	CPG_2685	78875861	78875863	rs7178270	78921077	-45216	-0.1	3.9E-05
	CPG_2685	78875861	78875863	rs3743075	78909452	-33591	-0.1	1.6E-04
	CPG_3898	79006008	79006010	rs3743075	78909452	96556	0.1	3.0E-04
	CPG_959	78700327	78700329	rs7178270	78921077	-220750	0	4.3E-04
	CPG_2021	78809529	78809531	rs1948	78917399	-107870	-0.1	4.5E-04
	CPG_4132	79041167	79041169	rs3743075	78909452	131715	0.1	4.8E-04

Notes: 1) bp: base pair; Chr: Chromosome; Variant Position: SNPs position in chromosome; Distance: the distance from associated SNP to CpG site; SNP: single-nucleotide polymorphisms; Unadj: Unadjusted; 2) Age and FTND score as covariates to adjust the methylation level.
